# The Need for Targeted Prevention and Screening in Gastrointestinal Cancer Disparities

**DOI:** 10.1016/j.gastha.2025.100742

**Published:** 2025-07-10

**Authors:** Jason C. Lin, John C. Lin, Joy Zhao

**Affiliations:** Division of Gastroenterology, Hepatology, and Nutrition, University of Texas Health Science Center, Houston, Texas; Perelman School of Medicine, University of Pennsylvania, Philadelphia, Pennsylvania; Division of Gastroenterology and Hepatology, Thomas Jefferson University Hospital, Philadelphia, Pennsylvania

Dear Editor

We commend Tang and Ko for their insightful work examining the disaggregated incidence of gastrointestinal (GI) cancers among Asian American, Native Hawaiian, and Pacific Islander (AANHPI) populations.^1^ AANHPIs represent the fastest-growing minority population in the United States, yet disparities in cancer incidence remain underexplored, particularly at a community level. Their research is essential in highlighting these disparities and investigating potential etiological factors. Given their findings, we would like to propose directions for future research and public health interventions.

One area that warrants further investigation is the differential cancer risk between US-born and immigrant AANHPIs. Tang and Ko demonstrate that AANHPIs in the United States have different incidences of GI cancer compared to their counterparts abroad. Considering that immigrants often face greater barriers to health-care access, a key question is whether health-care disparities contribute to increased gallbladder cancer risk in this population.

Building on Tang and Ko’s findings, we suggest focusing future efforts on tailored strategies for both primary and secondary cancer prevention among high-risk AANHPI groups based on a well-established gastric cancer (GC) prevention framework.^2^

A key aspect of primary prevention involves reducing known cancer risk factors. Helicobacter pylori (Hp) eradication should be prioritized in accordance of American College of Gastroenterology guidelines,^3^ particularly for individuals with a family history of GC in a first-degree relative, first-generation immigrants from high-Hp-prevalence regions, and racial/ethnic groups at increased risk for GC. Ensuring confirmatory testing after treatment and considering precision antibiotic selection based on resistance patterns would strengthen eradication efforts. Additionally, broader population-based Hp screening programs should be explored if further US data supports feasibility. Public health interventions—including increasing awareness among health-care providers, implementing educational campaigns targeted at high-risk populations, and addressing antibiotic resistance concerns—would further enhance preventive measures.

In terms of secondary prevention, expanding appropriate cancer screening remains crucial. Upper endoscopy with biopsies should be offered starting at age 50 for individuals with a family history of GC or first-generation immigrants from high-GC-incidence regions such as East Asia, Eastern Europe, and Andean Latin America.^4^ For those diagnosed with gastric intestinal metaplasia, continued endoscopic surveillance should be considered, particularly for individuals with extensive or incomplete gastric intestinal metaplasia, a family history of GC, or racial/ethnic backgrounds associated with higher GC risk. Additionally, provider education initiatives should aim to improve awareness of screening guidelines among US-based gastroenterologists, close gaps in routine confirmatory Hp eradication testing, and expand access to endoscopic screening in high-risk communities.

Tang and Ko’s work lays a crucial foundation for understanding GI cancer disparities among AANHPIs. Future studies should expand on these findings by investigating the intersection of immigrant status, health-care access, and cancer risk. Additionally, targeted prevention and screening strategies tailored to high-risk AANHPI subgroups will be instrumental in mitigating disparities and improving cancer outcomes.
